# Optimization of Titanium Alloy Drilling to Minimize the Secondary Burr after Deburring Process

**DOI:** 10.3390/ma15238432

**Published:** 2022-11-26

**Authors:** Emilia Franczyk, Wojciech Zębala

**Affiliations:** Chair of Production Engineering, Faculty of Mechanical, Cracow University of Technology, 31-155 Cracow, Poland

**Keywords:** titanium alloy Ti6Al4V, drilling, burrs, deburring

## Abstract

The formation of burrs on the edges of machined surfaces is one of the inherent problems during machining. The burrs are formed both on the tool entry surface and exit surfaces. The paper proposes a modification of the drill involving an additional cutting insert located in the shank part. This innovative solution allowed the drill and deburring insert to be integrated, enabling both processes to be performed within one machining operation. The impact of the selected insert geometry and deburring process parameters on the size of burrs was determined during the experimental studies. Using the proposed deburring process for the Ti6Al4V titanium alloy, with appropriately chosen tool geometry and cutting parameters, reduces the secondary burr height by more than 90% compared to the original value.

## 1. Introduction

Industrial development brings about the necessity to apply newer and newer engineering materials and to improve their production and shaping processes. The measure of the progress in the production and machining processes is a continuous improvement of productivity with simultaneous reduction of costs, manufacture of products of increasingly better quality, and the use of sustainable development rules [1,2,3]. All this entails eliminating or minimizing the existing technological problems, such as burr formation on the edges during the machining process. The presence of burrs may cause difficulties or prevent the assembly of workpieces, reduce their strength, damage the adjacent parts, cause personal injury (e.g., cuts) or be a source of contaminations hindering the operation of machinery and equipment [4,5,6]. The problems occurring in connection with the burrs force the manufacturers to take appropriate actions. One of the applied solutions is the removal of burrs during separate operations (deburring). Thus, the following nomenclature is adopted: primary burr for burrs formed during the basic machining (drilling) process and secondary burr for remaining material after the deburring process. It must be noted that any additional deburring process extends the cycle time and increases the tool wear and labor intensity [4,7]. Consequently, productivity decreases, and the costs go up. Therefore, the burr formation minimization methods during the drilling process are most desirable.

The aerospace, automotive, and power generation industries, as well as the hi-tech sector, have increasingly high requirements in terms of machine parts quality [8,9]. The materials used there often need to be able to transmit large, often variable, mechanical and thermal loads and simultaneously have to be relatively lightweight and resistant to corrosion. Titanium alloys are becoming increasingly popular due to their unique properties [10]. However, they have one major drawback: poor machinability, forming the group S in the ISO material groups classification. Poor machinability means their ability to undergo machining is low, leading to technical problems, huge forces and vibration, high temperatures and strain in the cutting zone, and fast tool wear which in turn causes shape and dimension errors and poor workpiece surface quality [11].

The main reason for the burr formation is the plastic strain of material subject to the load from the cutting tool [5,7,12]. The phenomena which occur then include mainly material upsetting in the radial direction during the compression and bending and tearing off of the material during the chip formation and separation [12]. The authors indicate that the more plastic the material is, the more intensive burr formation is. In brittle materials, burrs are usually small, and material breaking on the machined edge is more likely to occur than burr formation [12,13]. 

It has been shown repeatedly that the primary burr formation can be significantly reduced by appropriately choosing tools and the machining conditions and parameters. Correctly understanding mechanisms and relationships allows the machining process to be optimized for the burr size criterion [6,7,14,15,16]. In the literature on the subject matter, there are attempts to minimize the burr formation by controlling the following factors: tool geometry and material, type and method of application of cutting fluids, process parameters (in case of drilling: cutting speed, feed rate), type of machined material or the sequence of operations [7,12]. Not all factors can be changed freely for obvious reasons. In addition, their impact depends on the type of operation and may be different for turning milling, drilling, etc. In the case of drilling, one of the most important factors affecting the burr size is the tool geometry. Increasing the point angle and the helix angle helps reduce the burr height and thickness [12]. In the case of titanium alloys, depending on the range of parameters, the feed rate impact can be nonlinear and of a heterogeneous tendency [16,17]. Increasing the cutting speed results in a bigger burr size, but this impact is small and usually insignificant [16,18]. There are also well-known interactions between the process parameters, making the optimal selection of the process parameters even more difficult [6,14,16,19].

Scientists and engineers have been searching for effective methods of deburring for years. The methods of mechanical, electrochemical, thermal deburring, and abrasive blasting are known. In addition, manual and automated methods are distinguished. The electrochemical deburring process is useful for removing burrs in hard-to-reach places, such as cracks and crevasses within a workpiece [20]. In such a process, burrs are exposed to a solution consisting of salt or glycol. An electrical current is then applied to the solution, which dissolves the burrs. Mechanical deburring involves using a machine to grind burrs off a workpiece [21]. Such a process forces the change of the drill to an abrasive tool, which extends the machining process. A variation of this method is the vibration grinding process [22]. An automated polishing/deburring process uses the toolhead with a pneumatic spindle in which a pressure sensor and a linear encoder are integrated [23]. A newer solution is the combination of the spherical deburring cutter and the custom-made tool path enables hole deburring on a 3D curved edge in a CNC machine, maximizing the tool life [24]. Finally, a successful attempt has been made to use cryogenic process cooling in order to reduce burrs [25].

Various authors have made attempts to develop or modify tools (drills) in order to minimize the primary burr formation or remove the burrs during the drilling process. For example, a modification of the HSS drill geometry involving the chisel edge removal, increasing the point angle, and increasing the core diameter brought beneficial results: a change of the burr type from a crown/transient burr to a uniform burr with a cap [26]. In another case, it was proposed to modify the cutting edge by changing its direction and degree of curvature, which reduced the axial force and changed the chip flow direction, helping to reduce the burr size [27]. Previous work attempted to introduce additional chamfer on the drill margin, which resulted in decreased height of exit burrs [28]. However effective, those attempts did not fully eliminate the existence of burr and were proven on a limited number of material types. Summarizing the above examples, it can be concluded that the modification of the drill or drilling process alone has limited effectiveness and may not be enough to reduce burrs to a significant extent. In response to this problem solution involving a movable connection of the cutting insert with the drill have been proposed [28,29,30,31,32]. However, they also have some disadvantages. As shown by Kim et al. [29], deburring efficiency of such a tool depends on the type of primary burr. Another issue is that the drilled hole is always (even if unwanted) being chamfered and worse, uncontrollably. Aside from this, such tools are complicated and consequently expensive in manufacturing and operation. 

To the best knowledge of the authors, research on the optimization of tools and deburring processes, especially for difficult-to-cut materials, has not been conducted to a sufficient extent so far. Accordingly, the authors of this article propose the method of mechanical deburring, which consists of using one complex tool. The paper presents a proprietary tool for minimizing the burr height during the drilling process. The novelty is using an additional insert in the shank part and applying the orbital drilling technique. Unlike grinding, using a tool with a defined cutting edge geometry allows for better control of the cutting process and a more efficient machining process. This technology has a number of advantages over many alternative methods such as brushing, electro-chemical deburring, and manual deburring. Among the most important advantages are high quality due to consistent deburring results, low costs for quality control, lower investment costs, and elimination of external deburring operations, including manual activities such as scraping or buffing down burrs using one or more tools, workpiece reclamping. Finally, the proposed method is highly automated and can be efficiently used in modern NC machining processes. An additional task involved studying the impact of edge geometry and selected machining conditions on the size of secondary burrs formed during the deburring process. The results showed that the use of the proposed tool for the machining of the Ti6Al4V alloy gives an over 94% reduction of the burr height in comparison with the standard drill and process. The solution presented in the paper has the potential to be applied in the industry, for example, in the automotive and aerospace industries. 

## 2. Materials and Methods

### 2.1. Workpiece Material

The tests were carried out for the Ti6Al4V titanium alloy using a flat bar, the dimensions of which are shown in Table 1. Chemical composition and basic mechanical properties of the alloy are presented in Table 2 and Table 3, respectively.

### 2.2. Characteristics of the Drill and Its Modification

**Figure 1:**
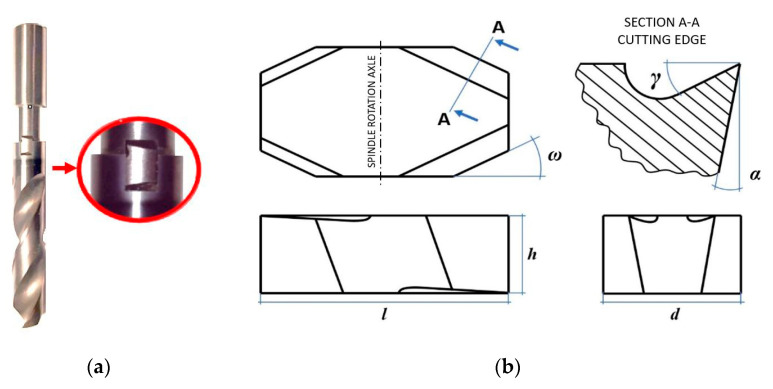
Drill with applied modification (**a**), geometry of deburring insert, length *l* = 9.8 mm, width *d* = 5.5 mm, height *h* = 3.2 mm (**b**).

The original tool was a Fanar X-Drill W9-604013-1000 3xD, a multi-purpose solid carbide twist drill designed to work with almost all types of materials and available in a wide range of diameters and lengths. Its diameter was 10 mm, and the point angle was 140°. The drill modification involved making a groove, a hole in the shank, and placing a proprietary cutting insert inside it, as presented in Figure 1a. The insert was made of cemented carbide by grinding the cuboid-shaped workpiece to obtain the correct shape, shown in Figure 1b.

**Figure 2:**
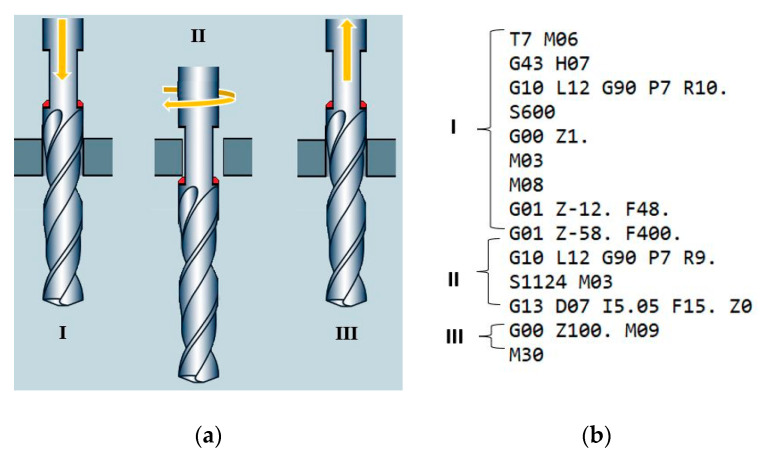
Integration of drill and deburring insert: three stages of the proposed process (**a**), machine code for drilling and deburring (**b**).

The cutting insert shape allowed its integration with the drill, consequently enabling the drilling and deburring in one operation without the need to change tools. An example of this solution and a fragment of the program implementing this operation is presented in Figure 2. As can be seen, the process consists of three main stages: (I) drilling, (II) positioning of the drill and performing orbital deburring, (III) retraction of the drill from the workpiece. During the second stage of the process, the tool rotates around its axis, and at the same time, it moves along a circle whose center is in the axis of the hole.

### 2.3. Experiment Design

**Figure 3:**
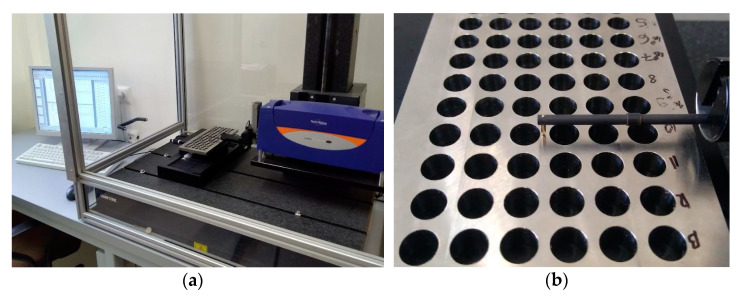
Burr height measurement stand (**a**), measured workpiece (**b**).

**Figure 4:**
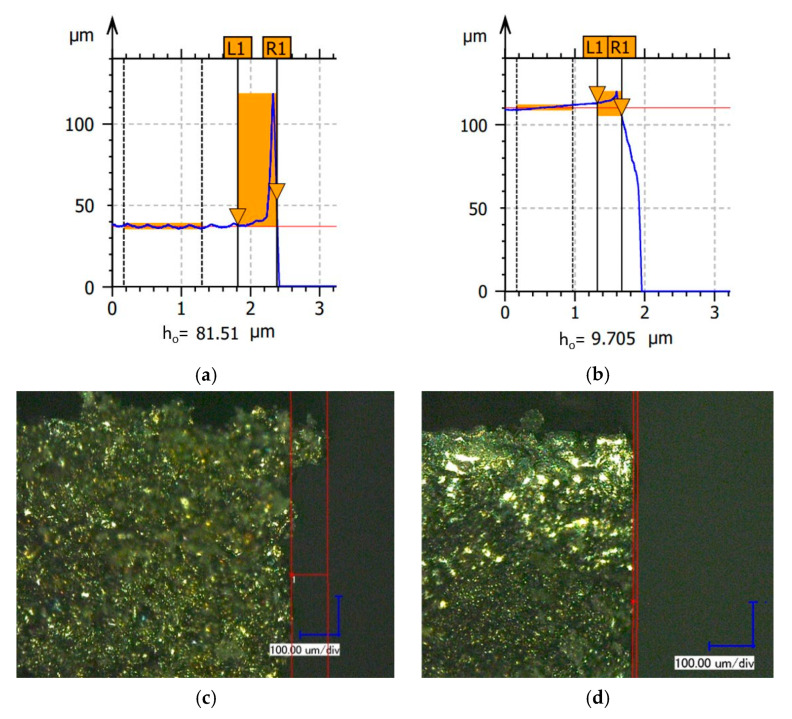
Burr height measurement result—primary burr (**a**,**c**) and secondary burr (**b**,**d**).

The test stand consisted of two main elements: the Haas Mini Mill machining center and the Taylor Hobson Talysurf Form 50 stationary profilographometer. The TalyMap computer software, supplied by the device manufacturer, was used for the acquisition and analysis of measurement data. Photographs of the measuring station and the measuring element are shown in Figure 3a,b, respectively. Figure 4 presents a printout containing exemplary measurement results (*h_o_*—the height of burr).

**Figure 5:**
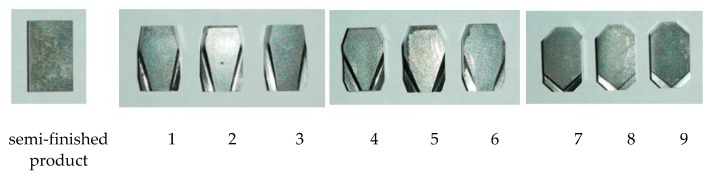
Pictures of cutting inserts. Designations according to Table 4.

The following parameters were used in the experimental studies: length *l*, width *d*, height *h*, and clearance angle α, which were 9.8, 5.5, 3.2 mm, and 8°, respectively. Three different values of tool rake angle γ were used in the experiments (8, 16, 24°), and three values of angle ω (10, 20, 45°). Nine inserts were made with different γ and ω parameters (Table 4). A photograph of all inserts is shown in Figure 5.

**Figure 6:**
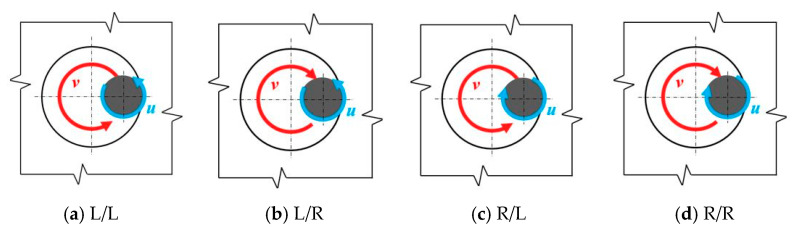
Types of kinematic systems (view from the bottom).

The deburring process consisted in processing the tool-exit edge of the holes with the use of the additional orbital movement of the drill. The tests were carried out with a constant feed of *f* = 0.08 mm/rev. The process variables were: the direction of the spindle rotation u [Right/Left] and the direction of the spindle orbital motion v [Right/Left], according to the diagram shown in Figure 6. In the further part of the work, the presented kinematic systems will be designated as L/L, L/P, P/L, and P/P, respectively.

The ranges of the independent variables tested are shown in Table 5. 4 samples were made for each test configuration.

## 3. Results

**Figure 7:**
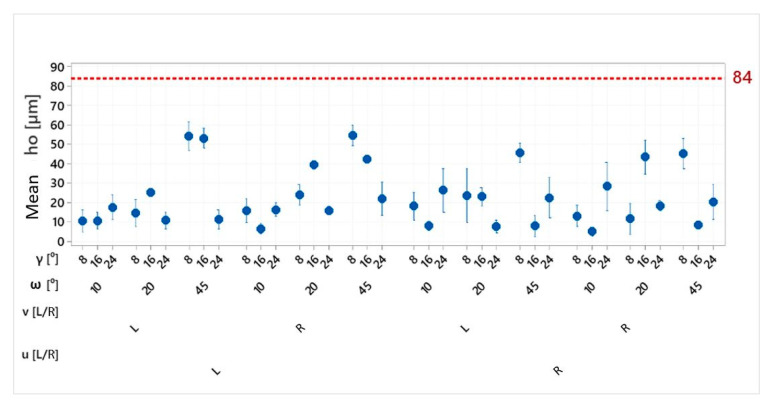
Results of measurements of the *h_o_* value of holes made with a modified drill.

The summary results of secondary burr height measurements after the integrated drilling and deburring operation are presented in Figure 7. The reference burr height (red broken line) was the mean height value of primary burrs obtained in a dozen drilling trials using the same drill but without the deburring process.

The analysis of obtained results indicates that in all trials, the burr size was reduced relative to the conventional process, and the obtained burr heights depended on the process parameters. Depending on the set of input parameters, the burr height was reduced by 35% to 94%.

The statistical analysis was performed independently for each kinematic case (Figure 6). The regression function was a second-degree polynomial described by the formula:

(1) $$h_{o}=\;A\cdot \gamma \cdot \omega \;+\;B\cdot \omega^{2}+\;C\cdot \gamma^{2}+D\cdot \omega \;+\;E\cdot \gamma \;+Z,$$

The equation coefficients were determined using regression analysis, and their statistical significance was determined using the ANOVA (Analysis of Variance) method. The results obtained in this way are summarized in Table 6. Significant factors in the equation (95% CI) are shown in bold.

**Figure 8:**
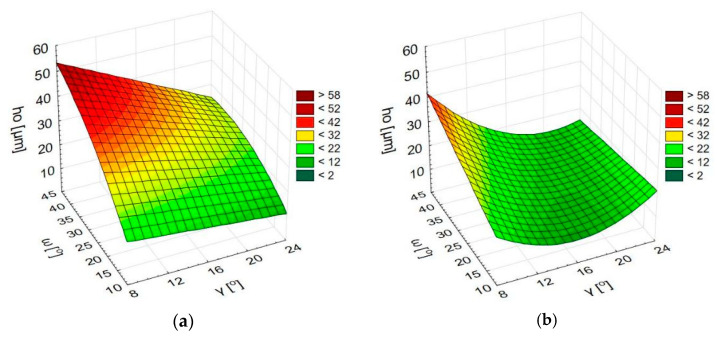

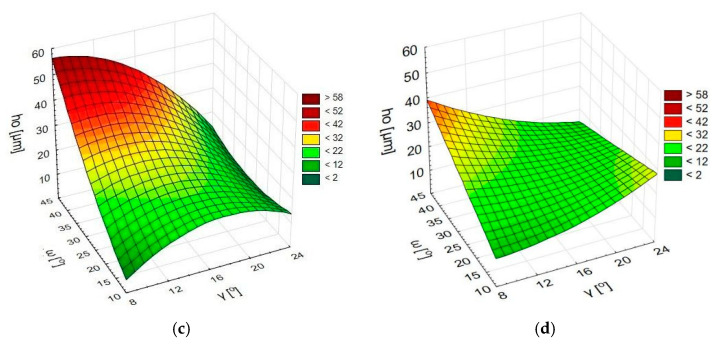
The height of burr *h_o_* as a function of the angles ω and γ; L/R process (**a**), R/L process (**b**), L/L process (**c**), R/R process (**d**).

Figure 8 contains 3D plots showing the height of the burr as a function of the angles ω and γ, made on the basis of the obtained regression functions. The charts were prepared independently for all kinematic cases.

Interactions between angles ω and γ were noticed in all observed cases. The impact of angle ω on the burr height is the greatest for small values of angle γ, and conversely, the impact of angle γ is the greatest for large values of angle ω. In each case, the greatest ho values were obtained for ω = 45° and γ = 8°, and the least values were obtained for small values of ω. In this case, the impact of angle γ is less significant and differs depending on the process kinematics. 

**Figure 9:**
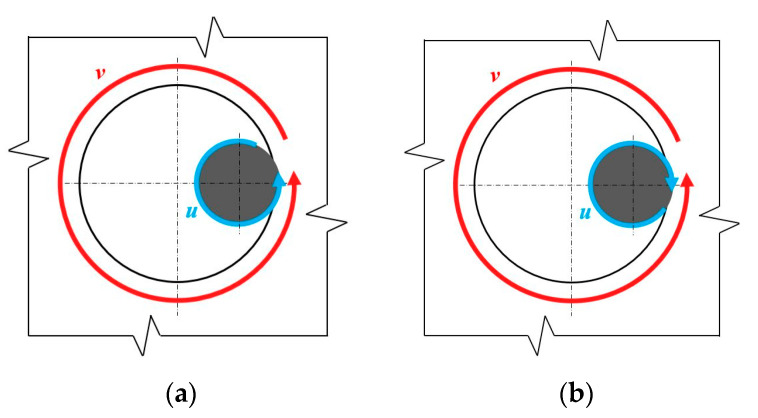
Types of relations between the insert kinematics (deburring) and the drill kinematics (drilling); common direction (**a**), opposite direction (**b**).

The impact of geometrical tool parameters was lesser in processes in which the spindle rotation was clockwise (R). The maximum burr height does not exceed 45 µm in this case, whereas in the processes with the anticlockwise (L) direction of rotation, the burr height reaches 60 µm. The difference between these processes may result from the relation of their kinematics to the kinematics of the drilling process used to make the preliminary holes (Figure 9).

In the case of the L/L and L/R processes, in the contact point, the direction of the cutting speed vector during the deburring is consistent with the direction of the cutting speed vector during the drilling. In the R/L and R/R processes, these directions are opposite. Assuming that a burr is formed, if only to a small degree, in the direction conforming to the drill rotation, it can be inferred that in the case of these two processes, the physical phenomena occur with a different intensity. In clockwise processes, the cutting edge encounters a larger resistance of the machined material, which can be a cause of higher temperatures, stronger vibration, and an increased share of friction forces. These factors may make the effect of the studied geometrical parameters less visible than in the case of anticlockwise processes. On the other hand, in the anticlockwise processes, the cutting edge slides on the burr surface and elastically pushes it into the machined material, resulting in a higher average burr height. More detailed studies are required to explain this problem in detail. Nevertheless, it is known that in order to obtain the smallest possible burrs, more attention is required when choosing the parameters for the L/L and L/R processes.

## 4. Discussion and Conclusions

The application of the integrated drilling and deburring process using the designed tool allowed a burr height reduction even by 94% in comparison with the conventional process and tool. In addition, it was proved that:

- The height of secondary burrs on the exit edges of holes made with the use of the proposed process depends on the cutting insert geometry, that is, on the angle between the cutting edge and the plane perpendicular to the spindle axis and on the insert rake angle, however the force of their effects depends on the deburring process kinematics.
- The least secondary burr heights are obtained for small values of the angle between the cutting edge and the plane perpendicular to the spindle axis.
- The effectiveness of the proposed method depends on the interrelation between the direction of tool rotation during the deburring process and the direction of the drill rotation during the drilling process. The most advantageous kinematic case was when the tool rotation direction during the deburring was opposite to the rotation direction during the drilling.

The proposed integration of the deburring and drilling processes eliminated the need to change the tool and the necessity for the workpiece re-referencing. The elimination of these operations increases productivity while reducing the costs and energy consumption of the production process.

## Figures and Tables

**Table 1:** Workpiece dimensions.

Width [mm]	Length [mm]	Height [mm]
235	100	9

**Table 2:** Chemical composition of Ti6Al4V titanium alloy, [%].

Ti	Al	V	Fe	C	N	O	H	Other
Balance	5.933	3.900	0.103	0.010	0.013	0.077	0.001	≤0.040

**Table 3:** Mechanical properties of Ti6Al4V titanium alloy.

Tensile Strength Rm [MPa]	Yield Strength R0.2% [MPa]	Elongation A [%]	Reduction of Area Z [%]	Hardness (HRC)
1000	900	15	41	30

**Table 4:** Geometric parameters of cutting inserts.

No.	Rake Angle γ [o]	Angle ω [o]
1	10	8.0
2	10	16.0
3	10	24.0
4	20	8.0
5	20	16,0
6	20	24.0
7	45	8.0
8	45	16.0
9	45	24.0

**Table 5:** Research plan.

Angle ω [°]	Rake Angle γ [°]	Direction of the Spindle Orbital Motion *v*	Direction of the Spindle Rotation *u*
10/20/45	8/16/24	R/L	R/L

**Table 6:** Regression equation coefficients.

Instance	A	B	C	D	E	Z
L/P	**−0.040**	**−0.016**	−0.072	**2.224**	2.936	−30.665
P/L	**−0.049**	0.006	**0.171**	**1.014**	**−4.918**	**41.964**
L/L	**−0.091**	0.014	−0.154	**2.248**	**6.392**	**−52.641**
P/P	**−0.073**	−0.026	**0.055**	**1.378**	−0.135	−14.074

## Data Availability

Not applicable.
